# Multiplex Flow Cytometry Barcoding and Antibody Arrays Identify Surface Antigen Profiles of Primary and Metastatic Colon Cancer Cell Lines

**DOI:** 10.1371/journal.pone.0053015

**Published:** 2013-01-07

**Authors:** Kumar Sukhdeo, Rosanto I. Paramban, Jason G. Vidal, Jeanne Elia, Jody Martin, Maricruz Rivera, Daniel R. Carrasco, Awad Jarrar, Matthew F. Kalady, Christian T. Carson, Robert Balderas, Anita B. Hjelmeland, Justin D. Lathia, Jeremy N. Rich

**Affiliations:** 1 Department of Stem Cell Biology and Regenerative Medicine, Cleveland Clinic Lerner Research Institute, Cleveland, Ohio, United States of America; 2 Department of Pathology, Case Western Reserve University School of Medicine, Cleveland, Ohio, United States of America; 3 BD Biosciences, San Diego, California, United States of America; 4 Department of Pathology, Brigham and Women's Hospital, Boston, Massachusetts, United States of America; 5 Department of Colorectal Surgery, Cleveland Clinic Digestive Disease Institute, Cleveland, Ohio, United States of America; 6 Department of Cancer Biology, Cleveland Clinic Lerner Research Institute, Cleveland, Ohio, United States of America; 7 Department of Cell Biology, Cleveland Clinic Lerner Research Institute, Cleveland, Ohio, United States of America; University of Chicago, United States of America

## Abstract

Colon cancer is a deadly disease affecting millions of people worldwide. Current treatment challenges include management of disease burden as well as improvements in detection and targeting of tumor cells. To identify disease state-specific surface antigen signatures, we combined fluorescent cell barcoding with high-throughput flow cytometric profiling of primary and metastatic colon cancer lines (SW480, SW620, and HCT116). Our multiplexed technique offers improvements over conventional methods by permitting the simultaneous and rapid screening of cancer cells with reduced effort and cost. The method uses a protein-level analysis with commercially available antibodies on live cells with intact epitopes to detect potential tumor-specific targets that can be further investigated for their clinical utility. Multiplexed antibody arrays can easily be applied to other tumor types or pathologies for discovery-based approaches to target identification.

## Introduction

Colon cancer ranks among the most common cancers in terms of both cancer incidence and cancer-related deaths in Western countries [1]. Early-stage colon cancer can be managed successfully by surgical resection; however, metastatic disease is often refractory to treatment and responsible for the majority of morbidity and mortality. Clinical decision-making is guided by the American Joint Committee on Cancer TNM (tumor-node-metastasis) staging that is imperfect for prognosis and does not predict response to therapy. A critical need exists to identify objective markers of malignancy that could be used for early detection, prognostication, intervention, and/or targeting of cancerous cells. As an example, tumor-associated antigens (TAAs) have been utilized for the detection of circulating tumor cells (CTCs) in the bloodstream as well as disseminated tumor cells (DTCs) in the bone marrow with the ability to monitor tumor burden, predict risk of progression, and measure chemotherapy response. These technologies include clinically-approved products such as CellSearch™ (Veridex) that utilize immunodetection of CTCs on the basis of Epithelial Cell Adhesion Molecule (EpCAM) membranous expression [2]. Cell-surface TAAs in particular are also valuable because they are accessible to systemically-delivered targeting molecules (e.g. antibodies, aptamers, etc.) that could be used to deliver bioactive payloads, block signaling, or activate antibody-dependent cell-mediated cytotoxicity.

Efforts to identify TAAs in colon cancer and other cancer types have relied on a multitude of techniques, each with its own set of advantages and limitations [3], [4]. Gene expression microarray profiling or tumor-derived cDNA expression libraries with patient sera (e.g. Serological Identification of Recombinantly Expressed Clones, SEREX) are based upon RNA-level expression. These approaches can be problematic because post-transcriptional (e.g. miRNAs) and post-translational mechanisms of regulation exert significant influence over the actual amount of protein possessed by each cell along with the signaling function within the cell [5], [6]. That is, cells with low-level transcripts can contain disproportionately high levels of translated protein (e.g. long half-lives) and vice versa. Mass spectroscopy and protein microarrays arrays utilize whole-cell or fractionated lysates from colon cancer cells to detect differentially expressed TAAs. These protein-based methods to detect TAAs can be influenced by the inherent disruption of the natural protein conformation during sample preparation, predominant representation of intracellular proteins, and limited molecular resources (i.e. commercially available antibodies) to rapidly evaluate the potential of candidate biomarkers.

To identify TAAs, we performed a high-throughput immunophenotypic screening of primary and metastatic colon cancer using an antibody array containing near complete coverage of the cluster of differentiation (CD) surface molecule family as well as many other common surface antigens. We also multiplexed the antibody array screen through the use of fluorescent cell barcoding. Our strategy identified comprehensive surface protein profiles including TAAs shared across tumor samples as well as those that were disease state-specific. The pan-tumor TAA, integrin α6 (CD49f), was validated to have an expression profile similar to EpCAM, demonstrating the potential of this technology to identify candidate tumor biomarkers that could be used to further refine the detection of malignant cells, including CTCs/DTCs.

## Results

### Multiplex barcoding in combination with antibody array screening

Two primary adenocarcinoma (SW480, HCT116) and one metastatic (SW620) colon cancer cell lines were selected for our study. All three lines have an epithelial origin and their tumor biology has been well studied in the literature. SW480 was derived from a primary adenocarcinoma of the colon from a patient that subsequently relapsed with wide-spread mesenteric lymph nodes metastases that were used to derive the SW620 cell line [7]. The use of a patient-matched set of cell lines reduces genetic variability and allows for a more controlled comparison of the molecular changes following metastatic progression [8], [9].

**Figure 1:**
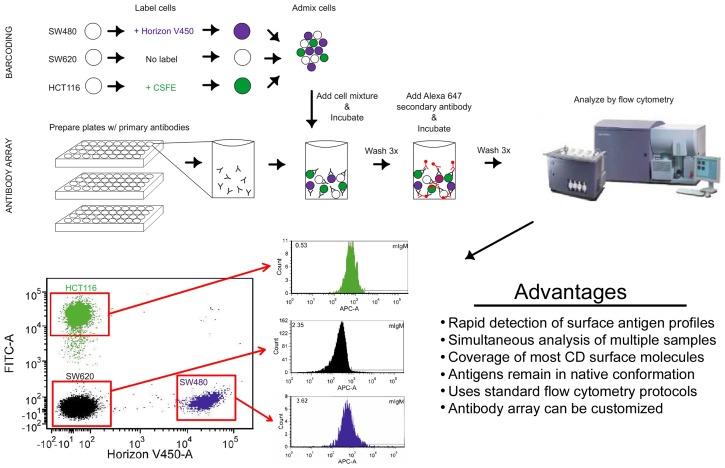
Diagram of experimental methods used for multiplexed barcoded antibody array. The three cell lines were labeled with or without intracellular dye prior to admixing the cells into a single pool. The cells were then aliquoted into each well for antibody labeling. The contents of each well were then processed on a flow cytometer. The identity of each cell line was determined based on fluorescence intensity. The appropriate gates were drawn allowing for simultaneous analysis for each antibody. Histograms for mouse IgM isotype control are shown.

Multiplexing of all three samples for simultaneous labeling and analysis was achieved through fluorescent cell barcoding. In this technique, cells are labeled with a distinguishing intracellular dye and then pooled together prior to antibody labeling. The identity of each cell line is recognized on the flow cytometer on the basis of fluorescence from either the violet (Horizon Proliferation Dye; VPD450) or blue (carboxyfluorescein succinimidyl ester; CFSE) excitation lasers, while the red laser is reserved for detection of Alexa647 on the secondary antibodies. The SW480 cell line was barcoded by labeling with VPD450 while SW620 cells were unlabeled prior to pooling both cell lines into a single admixed population (Figure 1, see Materials and Methods). The third cell line, HCT116, was barcoded using CFSE and also admixed to generate a single pool comprised of the three different cell lines. We then applied the combination of cells onto an antibody array consisting of 242 antibodies and 9 isotype controls individually allocated across three 96-well plates (Figure 1). The antibodies included in the array provide coverage of nearly every cluster of differentiation (CD) molecule and many other common surface antigens. As such, we were able to probe a broad range of surface proteins and generate signatures for each colon cancer cell line.

The majority of antibodies (158/242) were either completely unreactive or bound less than 5% of the cells as compared to the respective isotype control in all three cell lines and therefore not further investigated for the purposes of this study (full results shown in Figure S1 and Tables S1, S2, and S3). We found 25 antibodies that reacted with the majority (>50%) of cells in SW480, SW620, and HCT116 cell lines (Table 1 and Figure S2). Many of these antibodies reached near complete (>95%) labeling of tumor cells. As expected, proteins related to major histocompatibility complex class I (β2-microglobulin, HLA-A/A2/B/C and MIC-A/B) that are commonly expressed on nucleated human cells were identified. Other TAAs were categorized according to known function, which identified multiple proteins involved in extracellular matrix interaction and cellular adhesion, such as several integrin family members, in accord with their epithelial biology. Since α and β integrins form multimeric transmembrane complexes with each other, it is possible that the co-expression of integrin α2 (CD49b) and integrin α6 (CD49f) along with integrin β4 (CD104) may indicate functional interaction of these family members or shared regulation in colon cancer. We also identified several proteins with known function in cellular metabolism and signaling as well as others mediating interaction with the adaptive and innate immune system. These common identifiers could inform about tumor biology or represent druggable pathways to target tumor cells. Moreover, due to their broad expression on the surface of malignant cells, the pan-tumor antigens identified in this screen might be useful markers to facilitate the identification of CTCs/DTCs.

**Table 1:** Broadly expressed tumor antigens.

Antigen	Cell line (% Positivity)		
	SW480	SW620	HCT116
**β2-microglobulin** *	82.95	96.8	95.2
**CD9**	85.5	83.27	79.3
**CD44**	87.86	80.57	99.6
**CD46**	99.59	99.25	99.39
**CD47**	97.31	98.01	99.75
**CD49b/Integrin α2**	89.14	96.74	99.66
**CD49f/Integrin α6**	99.44	99.26	99.47
**CD58/LFA-3**	98.76	98.43	99.25
**CD59/MIRL**	99.76	99.66	99.42
**CD63**	84.62	97.03	99.0
**CD71/TFRC**	96.18	93.02	99.66
**CD81**	98.7	98.67	99.36
**CD97**	61.51	94.2	56.09
**CD98/LAT1**	86.54	85.68	81.19
**CD104/Integrin β4**	92.26	97.66	99.49
**CD146/MCAM**	62.15	64.14	91.83
**CD147/BSG**	99.7	99.74	100.0
**CD151**	69.32	87.31	85.71
**CD164**	87.3	90.86	98.14
**CD171/L1CAM**	69.09	83.99	58.07
**CD321/F11 Receptor**	65.46	93.87	95.79
**CD340/Her2**	58.32	76.38	93.9
**HLA-A,B,C** *	97.72	99.07	99.56
**HLA-A2** *	89.05	98.37	99.21
**MIC A/B** *	78.9	68.36	96.22

Antibody array results showing surface antigens that were expressed on at least 50% of cells in all three colon cancer cell lines analyzed. Arranged in alphanumeric order.

* , Antigens common to all nucleated human cells.

Abbreviations: lymphocyte function-associated antigen 3, LFA-3; membrane inhibitor of reactive lysis, MIRL; transferrin receptor protein 1, TFRC; large neutral amino acid transporter 1; LAT1; melanoma cell adhesion molecule, MCAM; basigin, BSG; L1 cell adhesion molecule, L1CAM; common leukocyte antigen, CLA.

### Bioinformatics analysis

**Figure 2:**
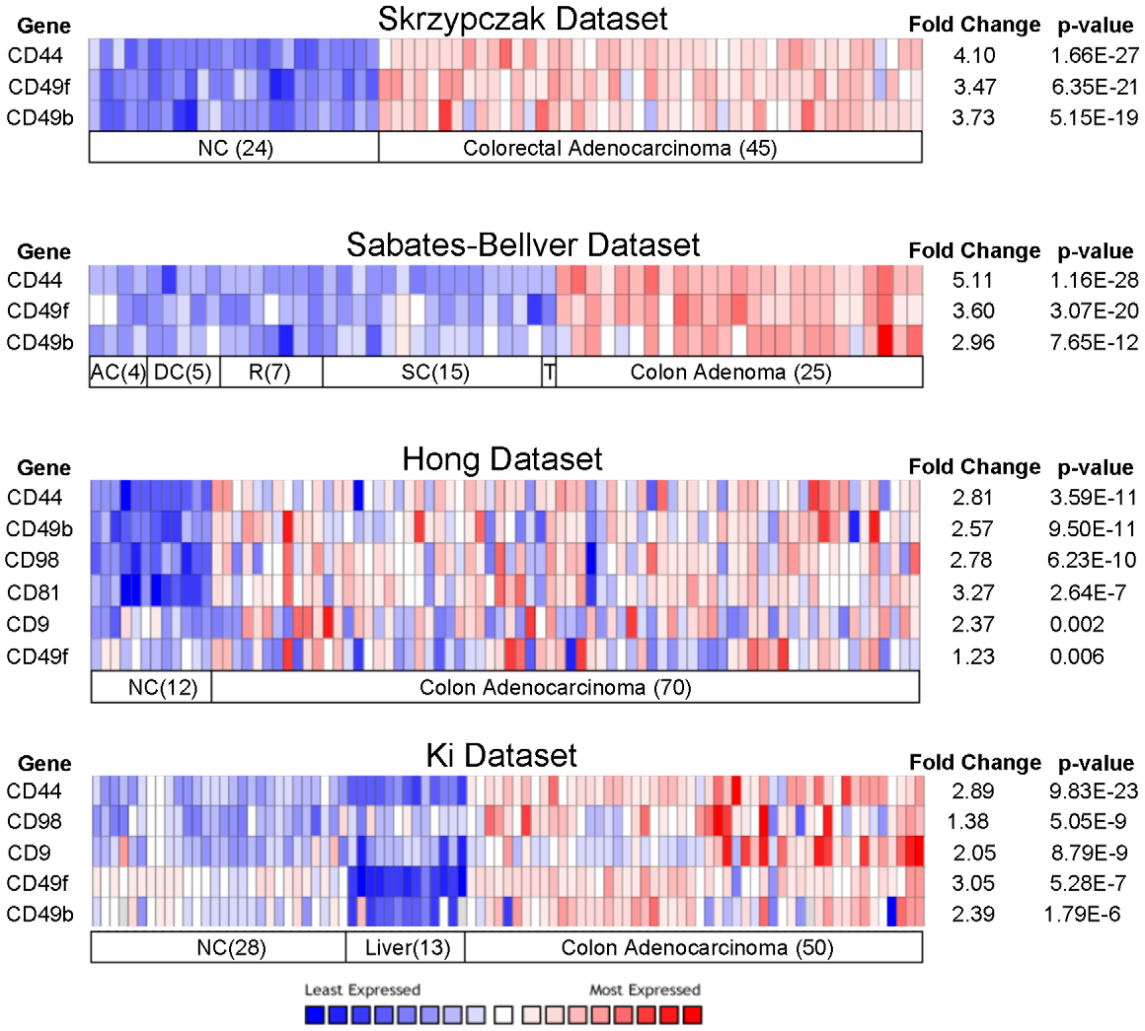
Oncomine analysis. Oncomine heatmap analysis in 4 published datasets for expression of tumor antigens described in Table 1. Only those genes that were consistently upregulated across datasets with p<0.05 are shown. The numbers in parentheses indicate the number of samples analyzed. Abbreviations: normal colon, NC; ascending colon, AC; descending colon, DC; sigmoid colon, SC; transverse colon, TC (n = 1).

To prioritize our list of TAAs as candidate biomarkers, we performed cross-comparisons to the Oncomine collection of gene expression microarray datasets from colon cancer as well as corresponding normal tissue [10]. As such, we were able to assess expression of the proteins identified in our screen as compared to RNA profiles across multiple investigators, patient populations, and experimental platforms. We focused our examination of TAA expression in normal colonic tissue, normal liver, as well as colon adenoma and adenocarcinoma [11]–[14]. We then selected those genes that were at least two-fold upregulated (p<0.05) over normal tissue. This further refined our TAA list to the overexpressed genes CD44, integrin α6 (CD49f), and integrin β2 (CD49b) as the most promising candidates (Figure 2). Notably, the expression of these genes was significantly higher in cancer than in normal liver, suggesting a possible therapeutic window for targeted therapies to spare normal tissue.

### Tumor marker validation

**Figure 3:**
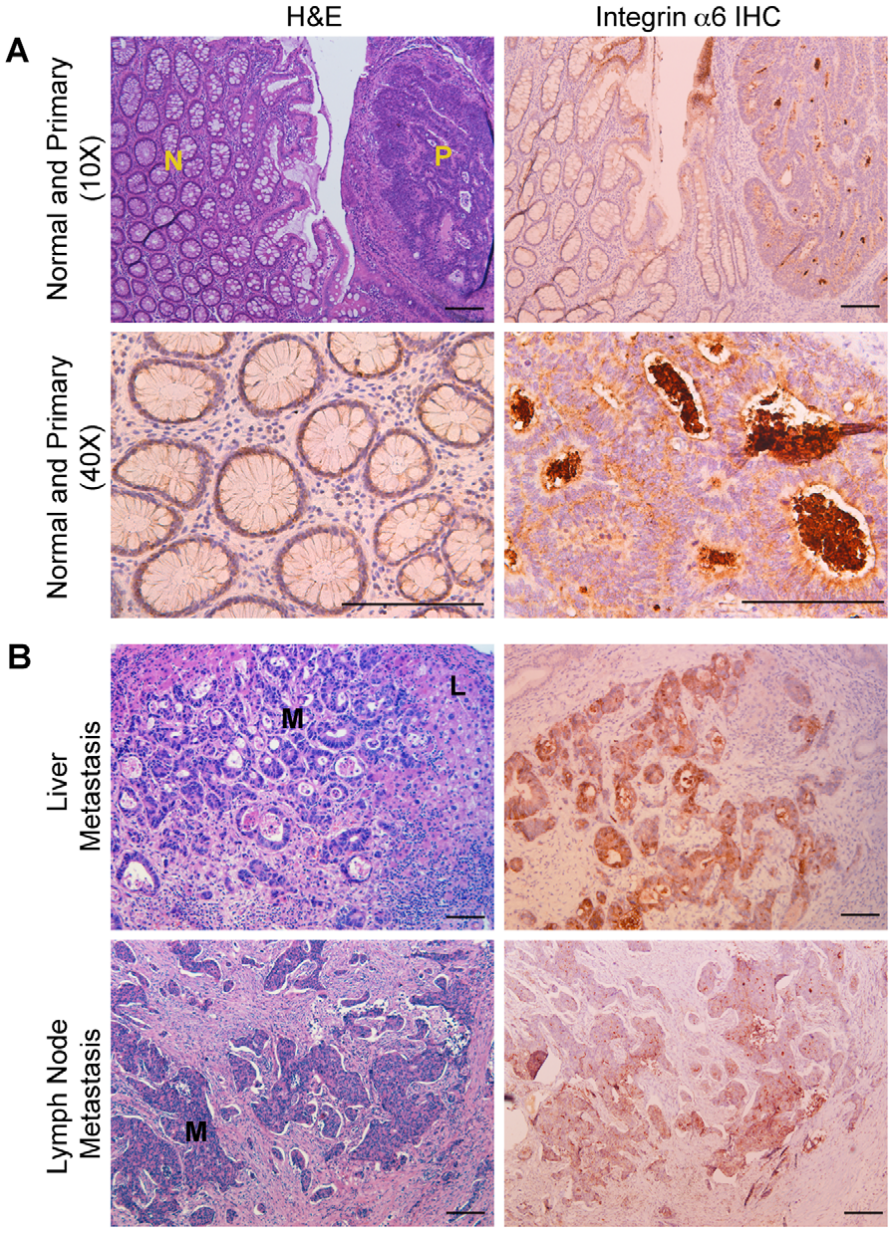
Validation of integrin α6 expression in colon cancer by immunohistochemistry. **A**) H&E (top left) and integrin α6 IHC (top right) from clinical colon cancer specimens at low magnification. Areas of normal mucosa (N) and adjacent primary colon cancer (P) are indicated. Lower panels provide higher magnification fields of integrin α6 in normal (left) and tumor (right). **B**) Representative examples of liver and lymph node metastases. The regions of colon cancer metastases (M) are visible by H&E (left) and corresponding staining with integrin α6 (right). An area of normal liver (L) is indicated. All lymph node samples contained a high degree of fibrosis around the lesion that displaced normal lymphoid tissue from the field of view. Scale bar: 50 µm.

To validate the results from our antibody screen to detect tumor cells in patient samples we performed immunohistochemistry (IHC) and immunofluorescence (IFC) staining on archived human specimens from normal colonic mucosa and primary colon cancer as well as metastases from liver and lymph nodes (n = 6 for each). We selected integrin α6 (CD49f) on the basis of its strong reactivity (>99%) with all three colon cancer cell lines in our screen, known expression in the intestine, and upregulation in tumorigenesis [15]. Indeed, by IHC, we could detect integrin α6 staining in colon cancer as well as in adjacent uninvolved normal mucosa. Moreover, all liver and lymph node metastases showed integrin α6 staining, whereas the surrounding stroma was negative. The difference in staining intensity between primary tumor and normal was subtle, but more pronounced in metastatic samples (Figure 3). These trends were also seen by IFC (Figure S3) using a distinct integrin α6 antibody in which co-labeling cells with epithelial marker EpCAM as a reference [16] showed that integrin α6 localized to all colon epithelial cells in every sample analyzed (Figure S3). These findings reinforce the utility of our screening method to identify TAAs that could be used for detecting tumor cells in patient samples and/or therapeutic targeting.

### Primary versus metastatic surface antigen signatures

**Figure 4:**
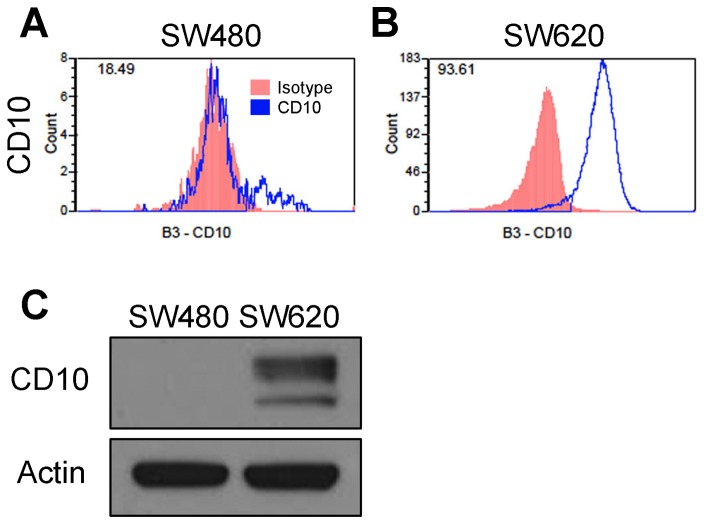
CD10 expression in SW480 versus SW620. Histogram plots from antibody array for the CD10 antigen in SW480 (**A**) and SW620 (**B**). Red indicates isotype control while the blue line is staining for CD10. The number in the top left is the cell positivity. There is a clear shift from a small shoulder population in SW480 to complete binding in SW620 cells. **C**) Immunoblotting for CD10 confirms the strong change in CD10 expression.

We next tested the ability of our antibody array screening method to compare and contrast the surface signatures from primary and metastatic disease by using SW480 and SW620 cell lines, respectively. Surface profiling identified 11 membrane-associated proteins that were present on at least 5% of cells and with at least two-fold increased cell positivity in SW620 as compared to SW480 (Table 2, Figure S4, and Table S4). CD10 (also known as membrane metallo-endopeptidase (MME)) had the highest fold-change in expression. It has enzymatic activity to degrade key signaling molecules and is upregulated in metastatic melanoma [17] (Figure 4A). The increase in expression of CD10 identified by the antibody was confirmed by Western blot, showing high level of protein in SW620 cells, but not in SW480 (Figure 4B). Interestingly, seven of the identified proteins have known roles in immune system function, suggestive of a role in immunomodulation during metastasis (Table 2). We also found 35 proteins with cell positivity reduced at least two-fold on SW620 cells as compared to SW480 (Table 3, Figure S5, and Table S4) including multiple representatives of proteins involved in cell metabolism/signaling, immune system signaling, and cellular adhesion.

**Table 2:** Antigens upregulated in metastasis.

Antigen	Cell line (% Positivity)		
	SW480	SW620	SW620/SW480 ratio
**CD10/MME**	18.49	93.61	+5.06
**CD119/IFNγR1** *	10.45	40.84	+3.91
**CD4**	21.44	69.40	+3.24
**CD109** *	20.92	64.00	+3.06
**CD43**	30.01	91.96	+3.06
**CLA**	17.80	52.50	+2.95
**CD205** *	11.42	32.24	+2.82
**CD227/MUC-1**	30.20	82.76	+2.75
**CD4v4** *	20.85	55.46	+2.65
**CD24**	36.73	91.06	+2.48
**CD99**	40.63	92.11	+2.27

Antibody array results showing surface antigens that were at least two-fold increased in cell positivity in SW620 (metastatic) as compared to SW480 (primary). Abbreviations: membrane metallo-endopeptidase, MME; cutaneous lymphocyte antigen, CLA; mucin 1, MUC-1.

* , Antigen was not two-fold increased by comparison of mean fluorescence intensities; however, differences in autofluorescence between cell lines limited the applicability of this analysis (Table S4).

**Table 3:** Antigens downregulated in metastasis.

Antigen	Cell line (% Positivity)		
	SW480	SW620	SW480/SW620 ratio
**EGFR**	51.50	1.12	−45.98
**CD21**	22.84	0.51	−44.78
**CD56/NCAM**	49.48	1.17	−42.29
**CD184/CXCR4**	17.55	0.62	−28.31
**CD73**	22.64	0.88	−25.73
**CD120b**	35.33	1.65	−21.41
**CD57**	94.71	5.10	−18.57
**CD162/SELPLG**	8.49	0.51	−16.65
**CD7**	6.31	0.51	−12.37
**CD70** *	7.19	0.59	−12.19
**CD87/uPAR**	7.33	0.63	−11.63
**CD3** *	5.79	0.70	−8.27
**CD95/FASR**	62.95	7.84	−8.03
**CD8b** *	6.55	0.82	−7.98
**CD33** *	5.76	0.77	−7.48
**CDw93** *	5.14	0.72	−7.14
**CD209/DC-SIGN**	10.76	2.16	−4.98
**CD45RA** *	5.83	1.22	−4.78
**CD91/LRP-1**	13.88	2.94	−4.72
**CD79b** *	7.32	1.62	−4.52
**CD153** *	11.70	2.65	−4.42
**CD130/gp130**	9.15	2.16	−4.24
**CD337/NCR3** *	7.32	1.74	−4.21
**CD100/SEMA4D**	6.60	1.58	−4.18
**CD193/CCR3** *	8.01	1.97	−4.07
**CD271/LNGFR**	76.08	19.47	−3.91
**CD181/IL8RA** *	5.07	1.31	−3.87
**CD6** *	8.09	2.10	−3.85
**CD243/P-gp** *	52.5	13.82	−3.80
**CD61/Integrin β3** *	5.22	1.51	−3.46
**CD75** *	8.54	2.55	−3.35
**CD107b** *	7.61	2.32	−3.28
**CD108**	7.16	3.22	−2.22
**CD54/ICAM1**	57.18	27.45	−2.08
**CD55**	73.75	36.77	−2.01

Antibody array results showing surface antigens that were at least two-fold decreased in cell positivity in SW620 (metastatic) as compared to SW480 (primary). Abbreviations: epidermal growth factor receptor, EGFR; neural cell adhesion molecule, NCAM; C-X-C chemokine receptor 4, CXCR4; selectin P ligand, SELPLG; urokinase receptor, UPAR; FAS receptor, FasR; dendritic cell-specific intercellular adhesion molecule-3-grabbing non-integrin, DC-SIGN; low density lipoprotein receptor related protein 1, LRP1; glycoprotein 130, gp130; natural cytotoxicity triggering receptor 3, NCR3; semaphorin-4D, SEMA4D; C-C chemokine receptor 3, CCR3; low affinity nerve growth factor receptor; LNGFR; interleukin 8 receptor alpha, IL8RA; P-glycoprotein, P-gp; intercellular adhesion molecule 1, ICAM1.

* , Antigen was not two-fold decreased by comparison of mean fluorescence intensities (Table S4).

### Expression of stem cell markers

To complete our surface antigen profiling of these colon cancer cell lines, we investigated the expression of membrane-associated cancer stem cell (CSC) markers. Notably, recent evidence has suggested that metastases are colonized by these CSCs that possess the functional abilities of self-renewal and multi-lineage differentiation [18]–[20]. CSCs may also function within tumors to propagate and/or maintain tumors over time and in response to treatment. Multiple groups have proposed various intracellular and extracellular identifying markers for CSCs that often have immunophenotypic similarities to normal tissue stem cells. Importantly, the detection of CSCs can be contingent upon antibody binding of post-translational (e.g. glycosylated) epitopes. The addition of such moieties to peptides often disconnects transcript levels from the amount detected by antibodies (e.g. *Prominin-1/CD133* transcripts and the CD133 (AC133) epitope in colon cancer CSCs [21]). Currently studied CSC surface proteins in colon cancer include EpCAM^high^, CD133, CD26, CD166, and CD44, independently or in combination [18], [20], [22]–[25]. CD26, a proposed marker of metastatic stem cells [20], and CD166 [24] were included in the antibody array and the results are provided in Figure S1. We performed standard multicolor flow cytometry for EpCAM, CD133, and CD44 to determine their expression individually as well as double and triple staining (Table 4 and Figure S6). Although we did not test the functional stem cell properties of any tumor cell populations, we were able to detect the varied expression of stem cell immunophenotype markers ranging from near absent to complete labeling. These results indicate that stem cell marker immunophenotypes can diverge greatly between tumors and is not always restricted to rare populations. Further characterization of these populations, beyond the scope of the current study, can determine the relevance of these expression patterns to functional phenotypes.

**Table 4:** Expression of surface stem cell markers.

	EpCAM+	EpCAM+CD44+	EpCAM+CD133+	EpCAM+CD44+CD133+
**SW480**	92.3	64.9	0.70	0.50
**SW620**	99.9	61.0	57.4	40.4
**HCT116**	95.8	95.3	85.3	85.2

Expression of putative surface cancer stem cell markers (% of live cells) in colon cancer cell lines as detected by multicolor flow cytometry.

## Materials and Methods

### Ethics statement

Normal colon and tumor specimens from patients treated at the Cleveland Clinic were obtained according to protocols approved by the Cleveland Clinic Institutional Review Board (IRB 4134), including written informed consent.

### Cell lines

Human colon cancer cell lines SW480, SW620, and HCT116 were all acquired from the American Tissue Type Collection (ATCC) [7], [8], [26]. Cells were grown in DMEM supplemented with 10% fetal bovine serum, 50 U/mL penicillin, 50 µg/mL streptomycin on standard tissue culture plates (BD Biosciences) in a humidified incubator at 37°C and 5% CO_2_. Prior to analysis, cells were in log-phase growth and <70% confluent. Detachment of cells from tissue culture plates was performed using TrypLE (Gibco) according to the manufacturer's protocol (10 minutes at 37°C). The Papain dissociation kit was obtained from Worthington Biochemical. Viability of cells was checked using Trypan blue exclusion and found to be >98%. All cells were grown and processed in parallel.

### High throughput flow cytometry analysis

High throughout flow cytometry analysis was performed on the three cell lines described above using an antibody screening method developed by BD Biosciences [27]. Antibody screening was done using the BD Lyoplate human cell surface marker screening panel (560747) containing lyophilized antibodies in a 96-well plate format at 0.5 µg/well. For flow cytometry analysis, the cell suspensions were treated with DNAse (in 1 ml PBS with Ca^2+^, Mg^2+^, 100 units/ml, 10 µl DNAse stock) for 15 minutes at room temperature. Prior to antibody staining, cell lines were barcoded with different viability dyes for simultaneous analysis [28]. SW480 cells were labeled with Horizon Violet Proliferation Dye 450 (BD Biosciences 562158) as per the manufacturer's protocol at 1 µM. HCT116 was labeled with CFSE (Invitrogen, C34554) as per the manufacturer's protocol at 1 µM. SW620 was left unlabeled and detected on the basis of being VPD450 and CFSE negative. Efficiency of labeling was >99%. After appropriate washing, all three cell lines were admixed and resuspended in BD Pharmingen Stain Buffer (BD Biosciences) with the addition of 5 mM EDTA to prevent reaggregation. Cells were plated into 96-well round bottom plates (BD Biosciences) at 30,000 cells (10,000 for each cell line) per well for staining. Cell surface staining was done with antibodies reconstituted with 1× PBS at a concentration of 0.5 µg per test and cells were stained live on ice for 20 minutes. Cells were washed thrice with staining buffer and then stained with species-specific Alexa647 secondary antibodies (BD Biosciences) for 20 minutes on ice. Cells were washed three times more in staining buffer, then resuspended in staining buffer with 7AAD (BD Biosciences) for live cell determination. Cells were analyzed on a FACS Canto system (BD Biosciences) equipped with a High Throughput Sampler (with plate loader) and data were compiled using with FlowJo software and a Microsoft Excel 2007 template from BD Biosciences (http://www.bdbiosciences.com/support/resources/stemcell/index.jsp#stemtools) for generation of heat maps.

### Immunohistochemistry

Tissues for IHC and IFC were obtained through a dedicated tissue procurement team within the Department of Anatomic Pathology at Cleveland Clinic. Samples for IHC were fixed in 4% phosphate-buffered formalin and embedded in paraffin wax for sectioning. IHC staining was performed on a Ventana Benchmark XT automated immunostainer utilizing a Ventana Optiview DAB IHC Detection Kit with CC2 antigen retrieval. Primary antibody, polyclonal rabbit anti-ITGA6, (Sigma-Aldrich, HPA012696) was diluted 1∶10.

### Immunofluorescence

Freshly harvested tumor or normal tissue was snap frozen and banked at −80°C. A gastrointestinal pathologist confirmed the histopathology diagnosis of each specimen independently. Normal tissue was obtained from a site distal from the primary colon tumor. Fresh frozen tissues were sectioned at a 6 µm thickness. Slides were fixed with 4% paraformaldehyde, air-dried, and stored at −20°C until use. After treatment with 10% normal goat serum and 0.1% Triton X-100 (Sigma-Aldrich) for 45 min, slides were incubated with monoclonal affinity purified mouse anti-human EpCAM (ab20160, Abcam) at a final dilution of 1∶200 and monoclonal affinity purified rat anti-human integrin α6 (MAB1378, Milipore) at a final dilution of 1∶100 overnight at 4°C, washed three times with PBS followed by incubation for 1 hour at room temperature with goat anti-mouse IgG1 AlexaFluor 568 (1∶1000 dilution) and goat anti-rat AlexaFluor 488 (1∶1000 dilution), both from Invitrogen. Slides were washed three times with PBS and counterstained with nuclear stain Hoechst 33342 (1∶10000) for 2 min. After washing with PBS, the slides were mounted with FluorSave (Calbiochem).

### Western blotting

Cells were lysed in 50 mM Tris pH 8.0, 120 mN NaCl, 0.5% NP-40, protease inhibitors (Sigma-Aldrich) and phosphatase inhibitors (Sigma-Aldrich). Equal amounts of cell lysate were loaded and resolved on a 4–12% bis-tris gradient gel (Life Technologies) and transferred to a PVDF membrane (Millipore). The membrane was simultaneously probed overnight at 4°C for anti-CD10 (mouse, 1∶500, Abcam), and anti-β-actin (mouse, 1∶8000, Sigma). Goat anti-mouse secondary antibody conjugated to horseradish peroxidase was detected using enhanced chemilluminescent substrate (1∶3000, Santa Cruz).

### Stem cell marker analysis

Additional antibodies for multi-color flow cytometry CD44-PE (Miltenyi; 1∶1000), EpCAM-FITC (BD Biosciences clone EBA-1; 1∶1000), and CD133-APC (Miltenyi AC133; 1∶1000). Cells were prepared as described above. FITC-conjugated isotype controls (Santa Cruz) were used separately for each antibody to determine baseline staining and compensation was performed according to standard techniques. For multi-color analysis, a single cell line was labeled with all three antibodies in a single tube, washed, and loaded onto a FACS Aria II flow cytometer (BD Biosciences). Gatings and plots were constructed using FlowJo software package.

### Oncomine analysis

Bioinformatics analyses were performed using the Oncomine database (www.oncomine.org). Genes of interest were evaluated based on a p-value cutoff of 0.05 and no expression level filter was used.

## Discussion

Improving outcomes for cancer patients will likely rely on new detection and treatment modalities for primary and metastatic disease. Here, we employed a novel high-throughput technique using a barcoded antibody array to define the surface antigen profiles of colon cancer cell lines derived from primary and metastatic tumor tissue. We identified a number of common and differentially expressed surface antigens, including those gained and lost in the transition to advanced disease (Tables 1, 2, and 3). The robustness of the system described herein arms investigators with the ability to screen global protein expression across disease states and/or the response of cells to particular stimuli. Among the applications of this approach, surface TAAs could be exploited for disease tracking and therapy. Some TAAs, such as EpCAM, have already entered the clinical realm with the ability to monitor disease burden. Additional TAAs should make it possible to add specificity and reliability in detecting tumor cells either in situ, in circulation, or as disseminated cells. Targeting of TAAs with monoclonal antibodies can also provide avenues to achieve tumor inhibition as has already been demonstrated in practice with cetuximab (Erbitux; against EGFR), rituximab (Rituxan; targeting CD20), and tositumomab (targeting CD20). HER2-positive advanced beast cancer can be inhibited by trastuzumab emtansine (anti-HER2 antibody coupled with a microtubule inhibitor) [29]. Moreover, radioimmunotherapy, such ibritumomab tiuxetan (targeting CD20), utilizes monoclonal antibodies to deliver radiation doses directly to tumor tissue.

Comparison of our candidate markers identified at the protein level via flow cytometry against RNA-based gene expression microarray databases of colon cancer (Oncomine) aided in our prioritization. However, inherent biological disconnects between RNA transcripts and outfitted polypeptides caution against using this filter as an ultimate determinant for selection of TAAs [5]. Rather, we favor the validation of informational content of TAAs on the basis of additional protein-level assays across a larger number of patient samples (e.g. immunohistochemistry on tissue microarrays). We validated integrin α6 in patient biopsies as a candidate tumor biomarker culled from our panel of antibodies. Further work will be necessary to address the clinical utility for integrin α6 and other identified surface antigens in tumor cell detection and therapy design.

Our results profiling human colon cancer cell lines expand upon those by Zhou et al. that also used a multiplexed antibody array [30]–[32]. Their study utilized a slide-based printed antibody array (DotScan™) with coverage of 122 cell surface markers. We found consistent signals with most, but not all, of their antibodies reacting with SW480 and SW620 cell lines, which may be attributable to sample preparation or analysis technique. In contrast to the DotScan antibody array, the antibody array used here probes nearly twice as many antigens using standard flow cytometry techniques available in most research facilities without the need for additional equipment or software (i.e. DotReader). The barcoding of cell lines can be further scaled up using 10-fold dilutions of intracellular dyes and/or double-labeled cells [28]. Similar to the DotScan method, however, our antibody array can also be multiplexed to analyze primary tumor samples containing multiple subpopulations that can be recognized by fluorescently conjugated antibodies (e.g. epithelial tumor cells with CEA-FITC and hematopoietic cells with CD45-APC) while the antibodies in the array are labeled with Alexa647. Alternatively, tumor subpopulations can be distinguished on the basis of physical (e.g. side population) or functional (e.g. stem cell assay) properties by labeling these cells at the expense of at least one fluorescent channel otherwise used for barcoding. For example, the Aldefluor assay is possible by labeling ALDH1-expressing stem cells in the green channel while sacrificing CFSE barcoding.

Several factors influence the surface profile of the cancer cells. Among these include the growth phase of cells, culture media, culture dish substrate, and the type of enzymatic detachment/dissociation, which can cleave epitopes. For example, treatment of HCT116 colon cancer cells with papain (enzyme used for dissociation of some solid tumors) reduced the detection of CD44 from 93.4% down to 0.5% of cells while EpCAM and CD133 (AC133) were not significantly affected (Figure S7). Thus, caution should be used when designing experiments and interpreting data from antibody-based screens. Additionally, our 5% cell positivity cut-off may omit rare, but biologically relevant cell populations and TAA biomarkers.

The combined barcoding and antibody arrays employed in the current study could be extended to rapidly profile additional tumor cells from colon and other tissue types. The ability to multiplex reactions reduces experimental variability, antibody consumption by 10- to 100-fold, and time to complete an assay. Moreover, this approach can be adapted for the simultaneous profiling of patient-derived normal, primary, and/or metastatic specimens in a single assay at a fraction of the time and expense. Lastly, the binding of known epitopes using commercially available antibodies expedites translational studies aimed at developing enhanced clinical resources.

## Supporting Information

Figure S1 **Complete antibody array results.** Complete results from barcoded antibody array screening of SW480, SW620, and HCT116 CRC cell lines. The position of each antibody on the plate is indicated from rows A–H and columns 1–12. To generate a heatmap of the expression of antigens, individual cells were colored on the basis of their expression value from 0 (white) to 100 (red). Note that the rat CD326/EpCAM in well F10 is only approved for mouse reactivity by the manufacturer and is a different antibody than that used in our immunofluorescence and multi-color flow cytometry.
(DOCX)

Figure S2 **Histogram plots from antigens in** **Table 1** **.** Antigens expressed in >50% of all cells in all three cell lines. Plot in red is corresponding isotype control. Blue line represents reactivity for the specified antibody.
(DOCX)

Figure S3 **Validation of Integrin α6/CD49f to identify CRC cells in patient samples.** Immunofluorescence was performed on normal colonic mucosa, primary CRC, liver metastases, and lymph node (LN) metastases. Representative examples are shown. Note increased intensity of staining near the basement membrane in cancerous tissue compared to normal. All tumor cells were readily identifiable in metastatic tissue whereas surrounding normal stroma was unreactive. All samples were processed and imaged identically. Inserts were imaged using confocal microscopy. Scale bar (150 µm). Inset scale bar (50 µm).
(DOCX)

Figure S4 **Histogram plots from antigens in** **Table 2** **.** Antigens with increase in percent positivity by at least 2-fold. Plot in red is corresponding isotype control. Blue line represents reactivity for the specified antibody.
(DOCX)

Figure S5 **Histogram plots from antigens in** **Table 3** **.** Antigens with decrease in percent positivity by at least 2-fold. Plot in red is corresponding isotype control. Blue line represents reactivity for the specified antibody.
(DOCX)

Figure S6 **FACS plots from stem cell marker analysis in** **Table 4** **.** Left: Histogram plots of EpCAM staining for the indicated cell lines. Red line indicates isotype control. Black line is reactivity for EpCAM antibody. Right: EpCAM+ cells from histogram gates shown on left stained with CD133-APC (y-axis) and CD44-PE (x-axis).
(DOCX)

Figure S7 CD44 antigen sensitivity to enzymatic detachment. Enzymatic treatment affects antigen expression. The HCT116 cell line was enzymatically detached from the tissue culture plate using either trypsin (TryPLE, left) or papain (right) prior to standard FACS antibody labeling and analysis. The expression of CD44 was virtually eliminated after papain treatment, suggesting enzymatic cleavage of this epitope.
(DOCX)

Table S1 Complete SW480 profiling results.
(XLSX)

Table S2 Complete SW620 profiling results.
(XLSX)

Table S3 Complete HCT116 profiling results.
(XLSX)

Table S4 Calculation and comparison of mean fluorescence intensities of SW480 and SW620 cells. Median, mean, and normalized fluorescence intensities derived from Tables S1 and S2. Fold change differences in SW480 and SW620 are calculated. Green shading: antigen was two-fold increased (SW620/SW480>2) by mean fluorescence intensity. Red shading: antigen was two-fold decreased (SW620/SW480<0.5). This list was then cross-referenced with the list of antigens identified by comparison of percent cell positivity in Tables 2 and 3. Discordance is indicated with an asterisk in Tables 2 and 3.
(XLSX)
