# Supplementary figures and images for: Multiplex Flow Cytometry Barcoding and Antibody Arrays Identify Surface Antigen Profiles of Primary and Metastatic Colon Cancer Cell Lines
Source: PLoS One. 2013 Jan 7;8(1):e53015. doi: 10.1371/journal.pone.0053015 (PMC3538639; doi:10.1371/journal.pone.0053015)

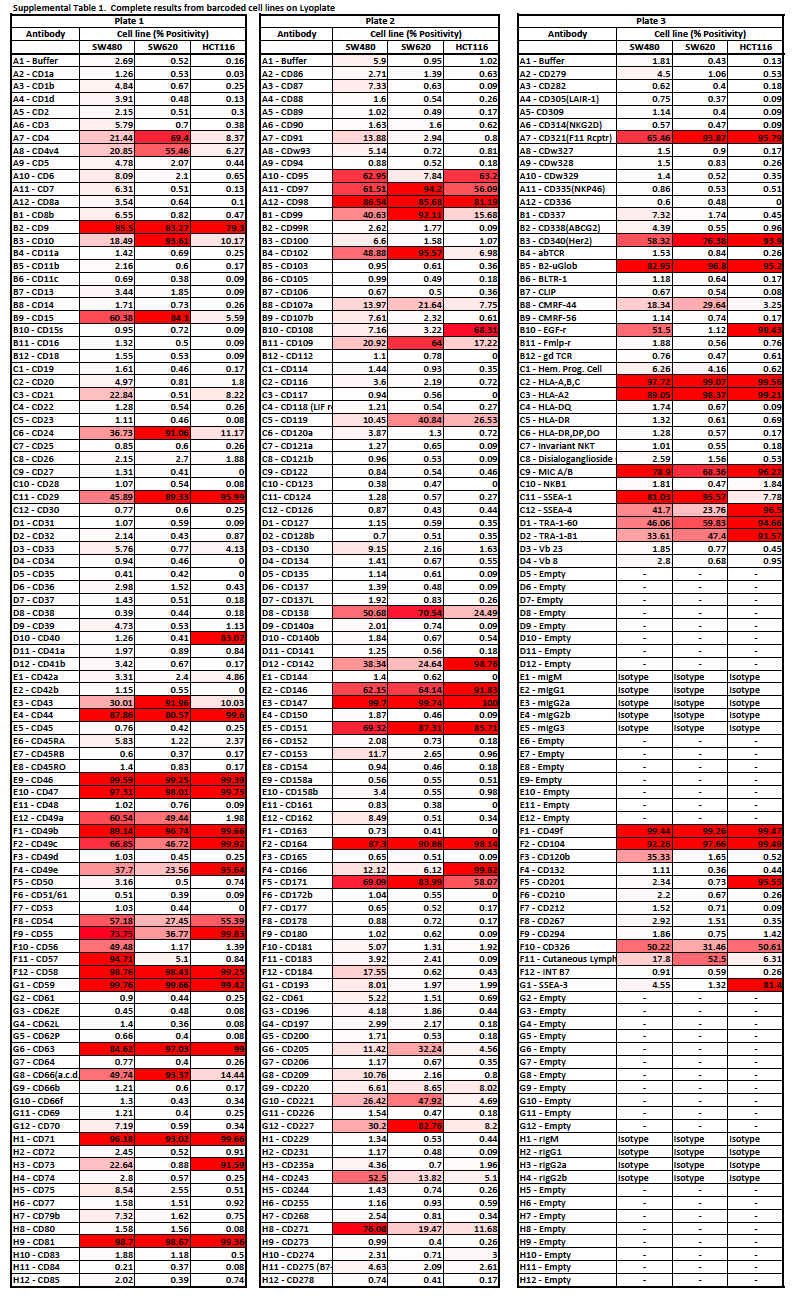

Supplement: Figure S1 — Complete antibody array results. Complete results from barcoded antibody array screening of SW480, SW620, and HCT116 CRC cell lines. The position of each antibody on the plate is indicated from rows A–H and columns 1–12. To generate a heatmap of the expression of antigens, individual cells were colored on the basis of their expression value from 0 (white) to 100 (red). Note that the rat CD326/EpCAM in well F10 is only approved for mouse reactivity by the manufacturer and is a different antibody than that used in our immunofluorescence and multi-color flow cytometry. (DOCX) [file pone.0053015.s001.docx]

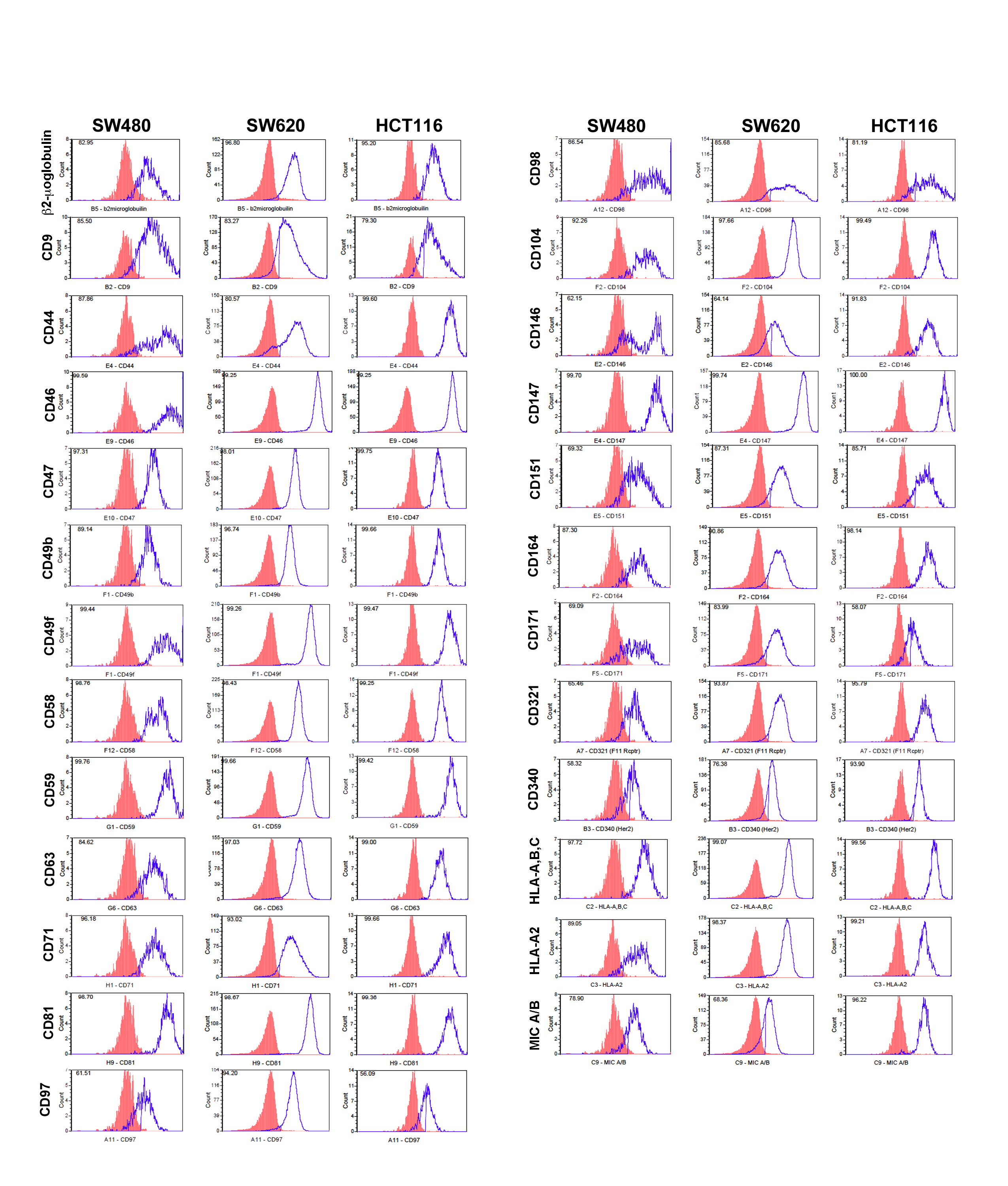

Supplement: Figure S2 — Histogram plots from antigens in Table 1 . Antigens expressed in >50% of all cells in all three cell lines. Plot in red is corresponding isotype control. Blue line represents reactivity for the specified antibody. (DOCX) [file pone.0053015.s002.docx]

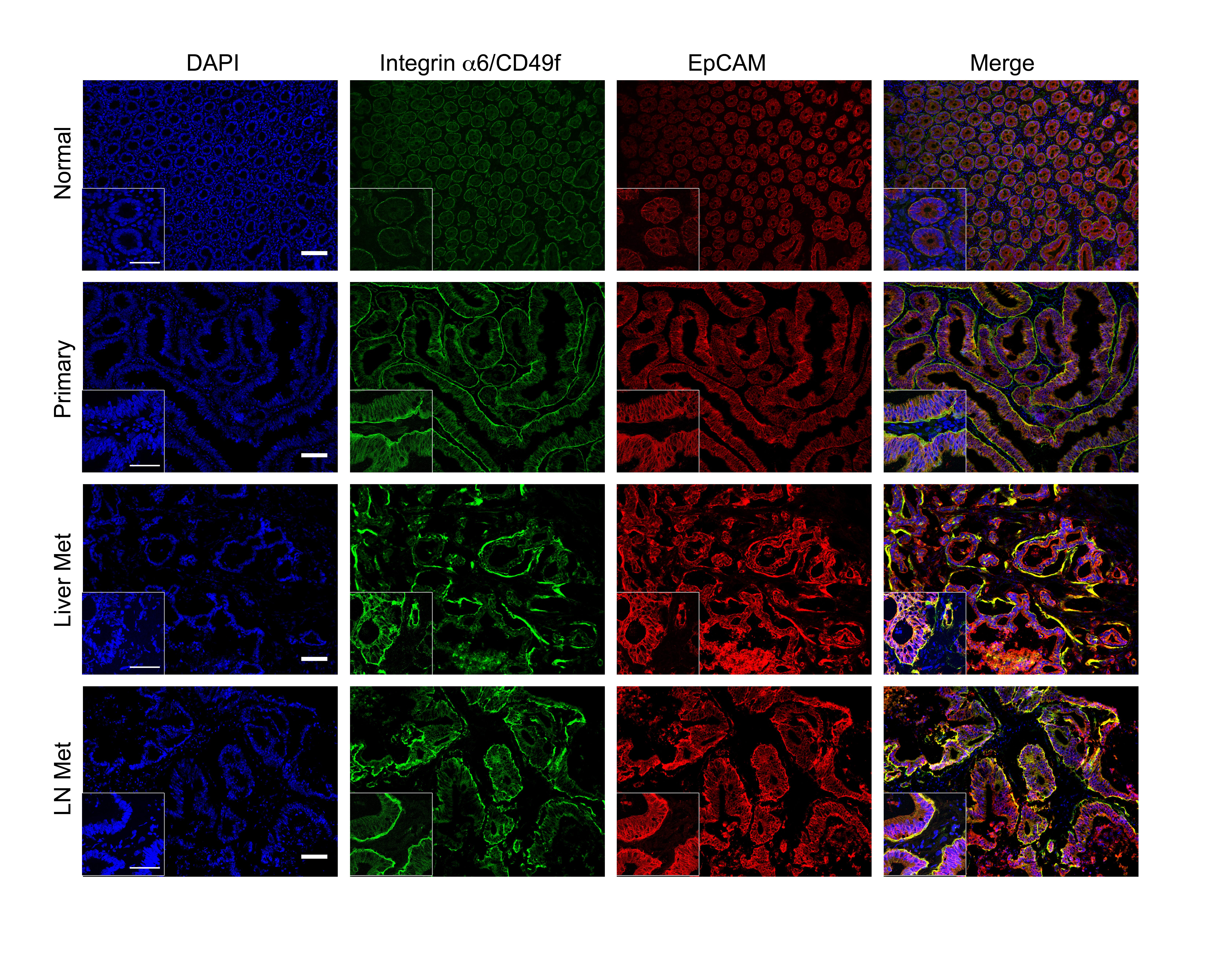

Supplement: Figure S3 — Validation of Integrin α6/CD49f to identify CRC cells in patient samples. Immunofluorescence was performed on normal colonic mucosa, primary CRC, liver metastases, and lymph node (LN) metastases. Representative examples are shown. Note increased intensity of staining near the basement membrane in cancerous tissue compared to normal. All tumor cells were readily identifiable in metastatic tissue whereas surrounding normal stroma was unreactive. All samples were processed and imaged identically. Inserts were imaged using confocal microscopy. Scale bar (150 µm). Inset scale bar (50 µm). (DOCX) [file pone.0053015.s003.docx]

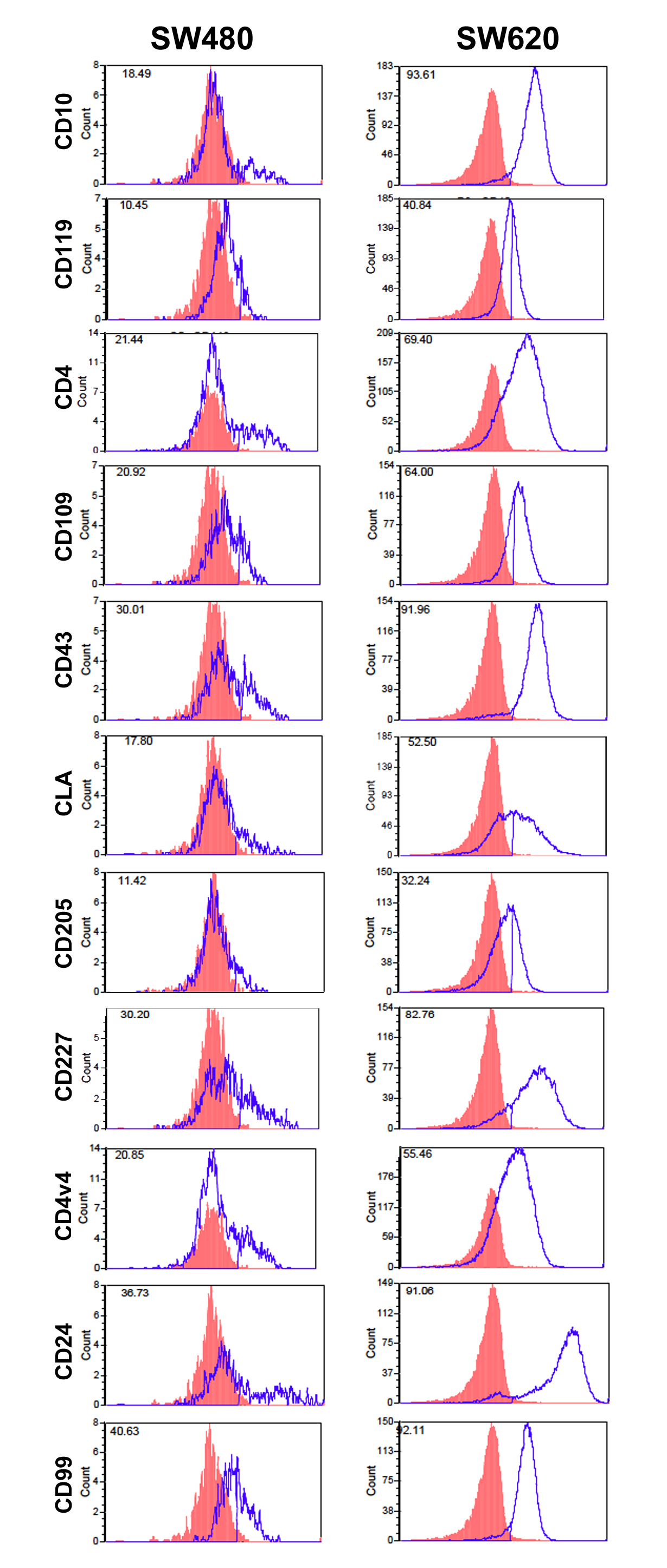

Supplement: Figure S4 — Histogram plots from antigens in Table 2 . Antigens with increase in percent positivity by at least 2-fold. Plot in red is corresponding isotype control. Blue line represents reactivity for the specified antibody. (DOCX) [file pone.0053015.s004.docx]

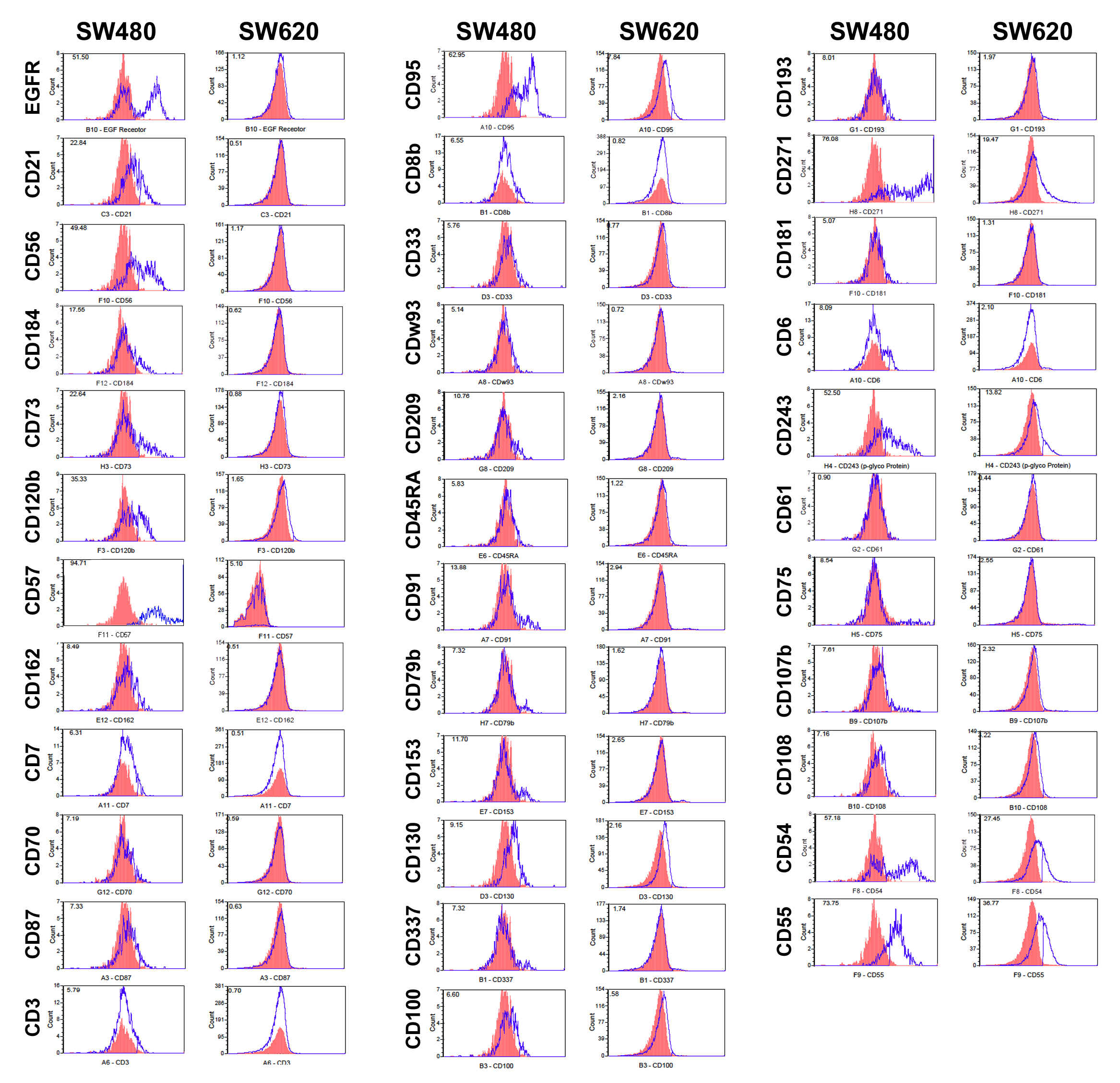
s

Supplement: Figure S5 — Histogram plots from antigens in Table 3 . Antigens with decrease in percent positivity by at least 2-fold. Plot in red is corresponding isotype control. Blue line represents reactivity for the specified antibody. (DOCX) [file pone.0053015.s005.docx]

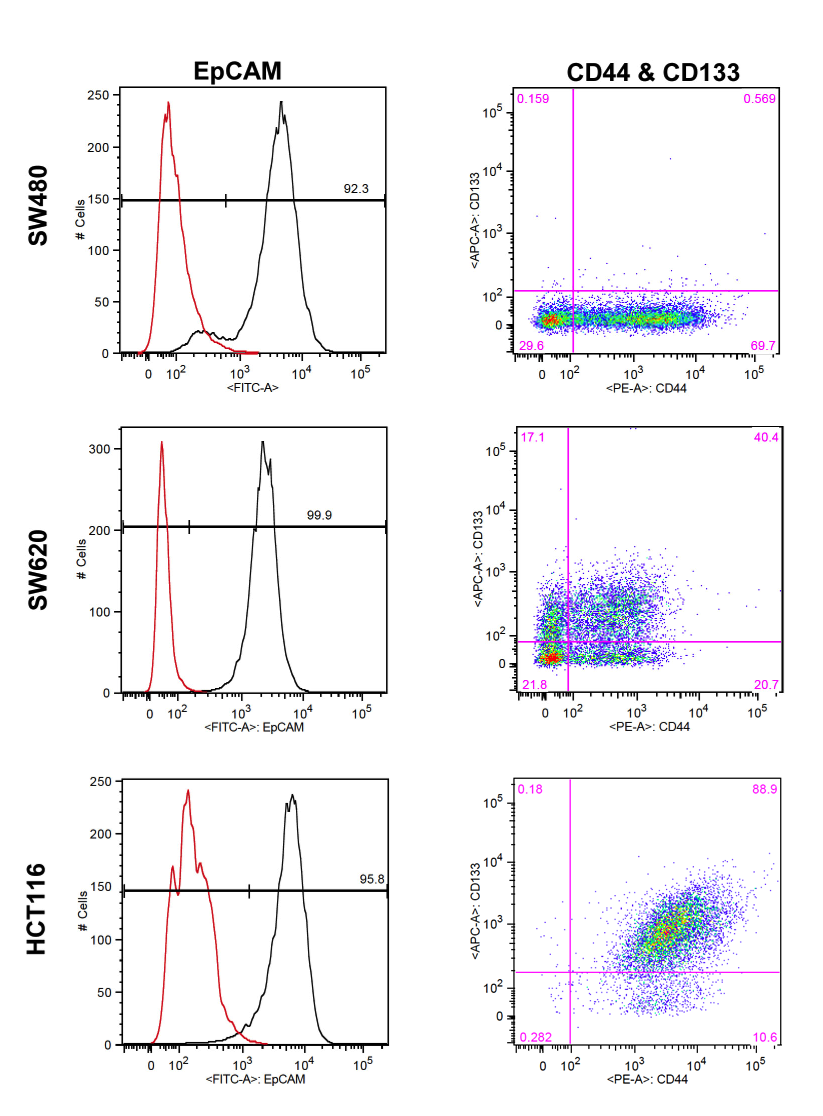

Supplement: Figure S6 — FACS plots from stem cell marker analysis in Table 4 . Left: Histogram plots of EpCAM staining for the indicated cell lines. Red line indicates isotype control. Black line is reactivity for EpCAM antibody. Right: EpCAM+ cells from histogram gates shown on left stained with CD133-APC (y-axis) and CD44-PE (x-axis). (DOCX) [file pone.0053015.s006.docx]

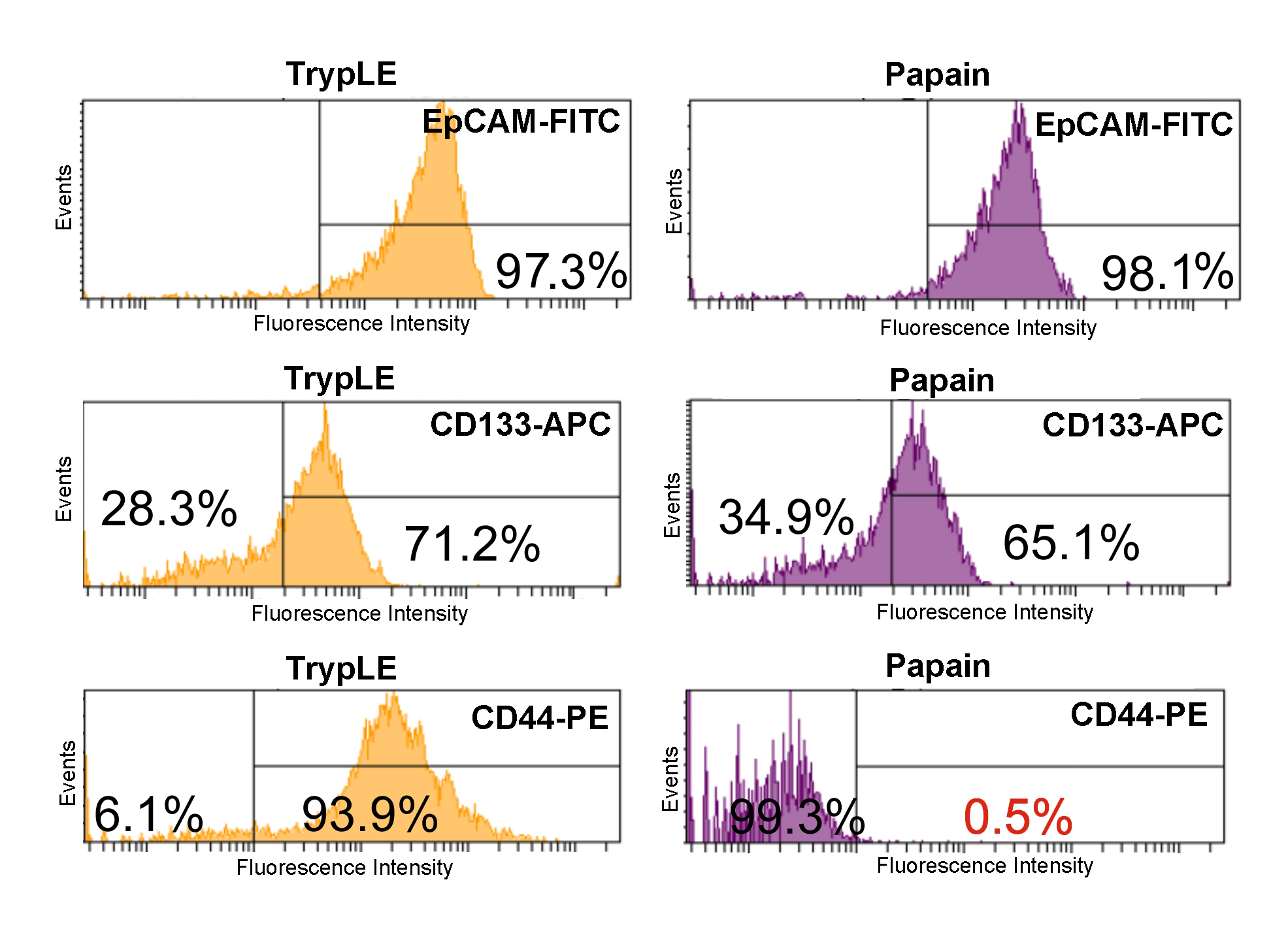

Supplement: Figure S7 — CD44 antigen sensitivity to enzymatic detachment. Enzymatic treatment affects antigen expression. The HCT116 cell line was enzymatically detached from the tissue culture plate using either trypsin (TryPLE, left) or papain (right) prior to standard FACS antibody labeling and analysis. The expression of CD44 was virtually eliminated after papain treatment, suggesting enzymatic cleavage of this epitope. (DOCX) [file pone.0053015.s007.docx]
